# Characterization of Conserved Combined T and B Cell Epitopes in *Leptospira interrogans *Major Outer Membrane Proteins OmpL1 and LipL41

**DOI:** 10.1186/1471-2180-11-21

**Published:** 2011-01-26

**Authors:** Xu'ai Lin, Aihua Sun, Ping Ruan, Zhe Zhang, Jie Yan

**Affiliations:** 1 Department of Medical Microbiology and Parasitology, School of Medicine, Zhejiang University, 388 Yuhangtang Road, Hangzhou 310058, PR China; 2 Basic Medical Microbiology Division, State Key Laboratory for Diagnosis and Treatment of Infectious Diseases, School of Medicine, Zhejiang University, Hangzhou 310058, PR China; 3 Faculty of Basic Medicine, Zhejiang Medical College, Hangzhou 310053, PR China; 4 Department of Medical Microbiology and Parasitology, Medical School of Shaoxing University, Shaoxing 312000, PR China

## Abstract

**Background:**

*Leptospira interrogans *are bacterial pathogens of animal that cause zoonotic infections in human. Outer membrane proteins of leptospire are among the most effective antigens which can stimulate remarkable immune responses during the infection processes, and thus are currently considered leading candidate vaccine antigens. The objective of the present study is to predict and confirm major combined B and T cell epitopes of leptospiral outer membrane proteins OmpL1 and LipL41, as well as to evaluate their capacity in the induction of immune responses in BALB/c mice.

**Results:**

In this study, four epitopes from OmpL1 and four from LipL41 conserved regions were evaluated for their potential utilization in leptospire vaccines. Firstly, combined B and T cell epitopes were predicted by softwares and expressed using a phage display system. OmpL1 residues 87-98 and 173-191 (OmpL1_87-98 _and OmpL1_173-191_) and LipL41_30-48_, LipL41_233-256 _of LipL41 were identified as immunodominant B cell epitopes by Western blot. Epitopes OmpL1_173-191_, OmpL1_297-320 _of OmpL1 and LipL41_233-256_, LipL41_263-282 _of LipL41 were identified as immunodominant CD4^+ ^T cell epitopes through proliferation analysis of splenocytes from recombinant OmpL1 (rOmpL1) or recombinant LipL41 (rLipL41)-immunized BALB/c (H-2^d^) mice. These epitopes induced responses of CD4^+ ^T cells and Th1 (T helper cells) type cytokine responses during the infection.

**Conclusion:**

This work identified combined T and B cell immunodominant epitopes in outer membrane proteins OmpL1 and LipL41 of *Leptospira interrogans*. OmpL1_173-191 _of OmpL1 and LipL41_233-256 _of LipL41 could be useful in a vaccine against *Leptospira*. The findings could also contribute to the development of effective cross-protective vaccine strategies for leptospirosis.

## Background

One of the emerging health problems in poor urban slum communities in developing countries is leptospirosis caused by pathogenic *Leptospira*, which is the most widespread zoonotic disease[1].

The immune responses to leptospires appear complex. Both animal model and human clinical studies have indicated that during the infection, leptospires can still persistently present despite robust immune responses suggesting that leptospires are capable of evading both innate and adaptive immunity and the immune responses triggered by leptospires in nature are not effective in the elimination of this pathogen [2]. Accumulating evidence support a key role for CD4^+ ^T cells in the acute and chronic stages of the infection in many bacterial diseases [3-5]. Immunity is specific for leptospiral types that have closely related agglutinating antigens, that is, the same or closely related serovars [6]. At present, the full genome sequences of some *Leptospira *strains have been sequenced [7-10], but the target antigens which are important in the induction of the host immune responses during infection have not been fully identified.

Leptospiral outer membrane proteins exposed on the leptospiral surface are conserved proteins among pathogenic *Leptospira *and are potentially associated with pathogenesis, and have become a major focus of current leptospiral vaccine research [11]. Some evidence has shown that outer membrane proteins play a critical role in the infection of *Leptospira*, because these proteins are at the interface between the pathogen and the mammalian host immune responses [12,13]. OmpL1 and LipL41 are antigenically conservative among pathogenic *Leptospira *species; their promise as vaccine candidates is enhanced by the finding that OmpL1 and LipL41 are expressed during infection of the mammalian host [14,15]. Recombinant outer membrane proteins OmpL1 and LipL41 were used as subunit vaccines and the protective effects were synergistic in a hamster model of leptospirosis [16].

In the present study, we expressed selected combined T and B cell epitopes of OmpL1 and LipL41 using a phage display system, and evaluated their ability of antibody recognition, as well as stimulation of T lymphocyte proliferation and cytokine expression.

## Methods

### Materials

*Leptospira interrogans *serovar Lai strain, used as the template in the amplification of epitope fragments, was cultured in EMJH medium. *Escherichia coli *DH10B was used as the host strain for the transformation. Phage display kit was purchased from New England Biolabs (Massachusetts, USA). Endonucleases, pGEM-T easy vector were obtained from Promega (Wisconsin, USA). 20-bp DNA ladder was bought from TaKaRa Bio (Dalian, China) Co., Ltd. Goat anti-human IgG-horseradish peroxidase (HRP) was from Jackson ImmunoResearch (Pennsylvania, USA), and goat anti-rabbit IgG-HRP was bought from Santa Cruz (California, USA). UltraEAL Western Blot Detection System was purchased from Shanghai Generay Biotech Co., Ltd (Shanghai, China). Protein molecular weight marker, lymphocyte separation medium (mouse), mitomycin and CCK-8 kit were purchased from Beyotime Institute of Biotechnology (Jiangsu, China). ELISA kits for IFN-γ or IL-4 were purchased from R&D Systems (Minnesota, USA). Sera from *L. interrogans*, recombinant protein OmpL1- or LipL41-immunized rabbits were produced as previously described [17,18]. Sera of leptospirosis patients were obtained from hospitals in Guangdong, Sichuan and Zhejiang provinces [19]. 6-8 week old female BALB/c mice were procured from the Experimental Animal Center of Zhejiang University and raised under pathogen-free environment. All the animal experiments were approved by the institutional review board.

### Prediction of T and B cell epitopes

The combined T and B cell epitopes were predicted based on the amino acid sequences of OmpL1 and LipL41 (GenBank accession codes AAT48511 and AAT48493). To avoid the epitopes located in the signal peptide region, SignalP 3.0 Server (http://www.cbs.dtu.dk/services/SignalP/) was used to predict the signal peptides. ANTIGENIC program in EMBOSS (http://beta.immuneepitope.org/) was used to predict B cell epitopes and ProPred, a web tool useful in the prediction of HLA-DR binding sites (http://www.imtech.res.in/raghava/hlapred/) [20], was used to predict potential T cell epitopes.

### Extraction of genomic DNA

The genomic DNA of *L. interrogans *Lai strain was extracted by proteinase K treatment and phenol-chloroform extraction method as described previously [21]. The precipitated genomic DNA was resuspended in 100 μl sterilized water, 10 μl 3 M sodium acetate and 220 μl absolute alcohol and stored at -20°C. Before use, DNA was precipitated by centrifugation and was resuspended in sterilized water.

### Expression and purification of epitope peptides

Sequences of 4 predicted epitopes from OmpL1 and 4 from LipL41 were amplified from genomic DNA. The primers used to amplify the fragments of selected epitopes were shown in Table 1. *Eco*R52 I site and a 14 bp leader peptide sequence of M13KE were located at the 5' end of each forward primer, and *Kpn *I was introduced at the 5' end of reverse primer. The amplified fragments were inserted into pGEM-T easy vector for sequencing. Then each of the sequence-confirmed fragment was subcloned into *Eco*R52 I and *Kpn *I sites of the phage vector M13KE. Primers M13PF 5'-GAGATTTTCAACGTGAAAAAATTATT-3' and M13PR 5'-TGAATTT TCTGTATGGGATTTTGCTA-3' were designed based on the sequence of PIII gene in M13KE and were used to determine the insertions of each epitope by colony PCR. Positive clones were further confirmed by sequencing.

**Table 1:** Primers for amplifying epitopes of OmpL1 and LipL41

Protein	Location	Primer	Sequence(5'-3')
OmpL1	59-78	O1-F59	cgGGTACCTTTCTATTCTCACTCTgttcgatcgtccaatacctg
		
		O1-R59	ttCGGCCGa**gccgcc**tgggttttgaaaacaagcag
	
	87-98	O1-F87	cgGGTACCTTTCTATTCTCACTCTtatataggagttgctcctag
		
		O1-R87	ttCGGCCGa**gccgcc**agcaggaatcgcttttctag
	
	173-191	O1-F173	cgGGTACCTTTCTATTCTCACTCTagttctatcgtcattcctgc
		
		O1-R173	ttCGGCCGa**gccgcc**agcgtcttcagtaacattc
	
	297-320	O1-F297	cgGGTACCTTTCTATTCTCACTCTctttctccttttccagc
		
		O1-R297	ttCGGCCGa**gccgcc**gagttcgtgtttataaccg

LipL41	30-48	L41-F30	cgGGTACCTTTCTATTCTCACTCTgtattcccgaaagataaaga
		
		L41-R30	ttCGGCCGa**gccgcc**acgaatggttccgaggaat
	
	181-195	L41-F181	cgGGTACCTTTCTATTCTCACTCTgtacgtatgatgttaattc
		
		L41-R181	ttCGGCCGa**gccgcc**tactttaatgagagtagc
	
	233-256	L41-F233	cgGGTACCTTTCTATTCTCACTCTgaagctgcttatatc
		
		L41-R233	ttCGGCCGa**gccgcc**tttaacgaaaactttaattc
	
	263-282	L41-F263	cgGGTACCTTTCTATTCTCACTCTaaagaacttcttcaagaaggtt
	
		L41-R263	ttCGGCCGa**gccgcc**ttttttgaaacttggagtttc

*Eco*R52 I and *Kpn *I sites are capital and underlined. Sequence encoding M13KE leader peptide is capitalized. The sequences in bold encode flexible peptide.

The proliferation and purification of phage was reported previously [22]. *E. coli *ER2738 was inoculated in 30 mL LB culture medium and incubated with shaking at 37°C for 2 h. Each recombinant phage was used to infect ER2738, and the culture was incubated with vigorous aeration at 28°C for 4 h. After centrifugation at 10 000 rpm for 10 min at 4°C, the medium supernatant containing phage was transferred to a clean tube and mixed with 1/6 volume of 20% polyethylene glycol 8000 (PEG 8000)-2.5 M NaCl and incubated at 4°C overnight. The phage was pelleted by centrifugation at 11 000 rpm for 15 min at 4°C and resuspended in 1 ml TBS (50 mM Tris-HCl, pH 7.5, 150 mM NaCl). The phage was reprecipitated by adding 1/6 volume of 20% PEG 8000-2.5 M NaCl and incubation on ice for 1 h. Finally, the recombinant phage was collected by centrifugation at 11 000 rpm for 15 min at 4°C and resuspended in TBS. The OD values at wavelength 269 and 320 were determined and used to calculate the number of phage particles according to the method of Day described previously [23].

### Identification of B cell epitopes

Western blot assay was used to detect the reactivity of B cell epitope with antibodies in the rabbit sera raised against *L. interrogans*, rOmpL1 or rLipL41. Purified recombinant phage particles (3 × 10^14^) were separated by electrophoresis in an 8% SDS-PAGE gel and then transferred to a polyvinylidene fluoride membrane (PVDF, Millipore). The membrane was blocked in 6% newborn bovine serum-TBST (Tris buffered saline; 0.1% Tween 20, pH 7.2) for 1 h and incubated overnight at 4°C with rabbit serum against *leptospira *Lai (dilution 1:200, MAT more than 1:400) followed by blotting with HRP-conjugated goat anti-rabbit antibodies (dilution 1:5000) for about 1 h at 37°C. Blots were developed using enhanced chemiluminescence reagents and exposed to X-ray films. In some cases, the blots were reprobed using rabbit serum against rOmpL1 or rLipL41(dilution 1:300) after stripping off the first antibody by incubation in the stripping buffer (65 mM Tris-HCl pH 6.7, 100 mM beta-mercaptoethanol, 2% SDS). Wild type M13KE particles were used as controls.

The reactivity of each epitope with antiserum mixture from IgM- and IgG-positive leptospirosis patients who were infected by different leptospiral serovars was also evaluated by Western blot [21]. IgM- and IgG-positive serum samples from leptospire-infected humans were pooled together and used as primary antibody (1:50 dilution). The antisera were incubated with the PVDF membrane at 37°C for about 1.5 h. After a washing step, goat anti-human IgG-HRP (1:5000 dilution) was used as secondary antibody. Wild type M13KE particles were also used as controls.

### Mice and immunization

100 μg rOmpL1 or rLipL41 protein pre-mixed with complete Freund's adjuvant (Sigma) was used to inject subcutaneously in the four limbs of 6-8 week old female BALB/c (H-2^d^) mice,. Same dose of proteins for boosting immune responses were given with incomplete Freund's adjuvant (Sigma) two weeks later. After 10 days, the mice were used for further experiments. Control mice were administered with PBS by the same procedures.

### Detection of T cell response

For the analysis of specific CD4^+ ^T cell proliferation, spleens were aseptically removed and mechanically homogenized with a 3-ml syringe plunger, and then splenocytes were isolated by lymphocyte separation medium (mouse) according to the manufacturer's instruction. Complete RPMI 1640 media (RPMI-1640, 10% fetal bovine serum, 2 mM glutamine, 50 U of penicillin/ml, 50 μg of streptomycin/ml, 50 μM 2-mercaptoethanol, and 25 mM HEPES) was used to cultivate the cells.

100 μl isolated T cells (5 × 10^4 ^cells per well) and mitomycin-inactivated allogeneic splenocytes (10^5 ^cells per well) were seeded into 96-well flat bottom culture plates and restimulated in vitro with different epitopes at a concentration of 5 × 10^14 ^recombinant phage particles per cell. 5 μg/ml mitogen concanavalin A (ConA) was used as control. Cells were incubated at 37°C with 5% CO_2 _for 72 h. The cell proliferation was measured using Cell Counting Kit (CCK)-8 according to the manufacturer's instruction. Briefly, 20 μl CCK solution was added to the culture medium and incubated for additional 3 h. The absorbance was determined at 450 nm with a 630 nm reference wavelength using ELISA Reader (Bio-Rad). Unstimulated cells were used as negative control and ConA-stimulated cells were used as positive control. Tests were repeated at least three times independently. Phages expressing each epitope were mixed together to evaluate if there is synergistic effect of these epitopes in the stimulation of the splenocytes isolated from the immunized mice.

### Effect of each epitope peptide on the secretion of cytokines

Th1 (IFN-γ) or Th2 (IL-4) cytokine secretion was examined. IFN-γ or IL-4 ELISA kit was used to evaluate the cytokine level in 100 μl T lymphocyte cell culture supernatants according to the manufacturer's instruction. Production of each cytokine was calculated through the titration of the supplied calibrated cytokine standards.

### Statistical analysis

Figures represent data from three independent experiments shown as mean ± SD. Microsoft office Excel was used to analyze variance and identify significant differences.

## Results

### Prediction and expression of combined T and B cell epitopes of OmpL1 and LipL41

The online softwares were used to map the combined B and T cell epitopes in OmpL1 and LipL41. Eight high-score combined T and B cell epitopes, including 4 OmpL1 epitopes and 4 LipL41 epitopes were selected as candidates for peptide expression and immunological analysis (Table 2).

**Table 2:** The sequences of selected epitopes from OmpL1 and LipL41.

Protein	Location	Amino acid sequence (N-C)
OmpL	158-78	VRSSNTCTVGPSDP**A**CFQNP
	
	87-98	YIGV**A**PRKAIPA
	
	173-191	SSIVIP**A**AVGIKLNVTEDA
	
	297-320	LSPFPAYP**I**VVGGQIYRFGYKHEL

LipL41	30-48	VFPKDKEGRAL**Q**KFL**G**TIR
	
	181-195	VRMML**IP**LDATLIKV
	
	233-256	EAAAYIKGRLSPI**V**KTERIKVFVK
	
	263-282	KELLQEGYEEI**V**GETPSFKK

The residues possibly anchoring MHC II molecular were underlined; the residues possibly binding B lymphocyte are bold.

**Figure 1:**
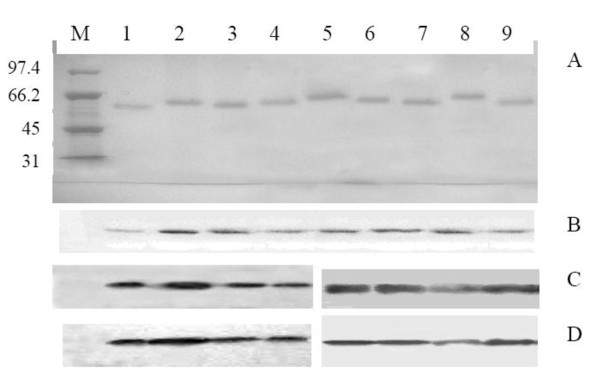
**SDS-PAGE and Western blot analysis of epitope-expressing phages**. 3 × 10^14 ^purified phage particles were separated by SDS-PAGE gel and transferred to PVDF membrane for Western blot assay. A is SDS-PAGE analysis of purified recombinant phage particles. B and C are the Western blot results, using rabbit sera against *Leptospira interrogans *or recombinant proteins. D is the result using sera mixture from five IgG- and IgM- positive leptospire patients. Lane M, protein ladder; lane 1, wild type M13KE particles; lane 2-5, recombinant phage particles containing epitope fragments 58-78, 87-98, 173-191 and 297-320 from OmpL1; lane 6-9, recombinant phage particles containing epitope fragments 30-48, 181-195, 233-256 and 263-282 from LipL41.

Each selected epitope of OmpL1 and LipL41 was first amplified from genomic DNA of Lai strain [Additional file 1], and then subcloned into the *Eco*R52 I and *Kpn *I sites of phage vector M13KE. The insertion of each epitope into the recombinant phage was confirmed by colony PCR [Additional file 2]. The sequences of the epitopes in the recombinant phage were confirmed via sequencing. Then the recombinant phage DNA was used to transform *E. coli *ER2738 competent cells. The recombinant phage particles were purified and separated on an 8% SDS-PAGE gel. Wild type phage M13KE was used as control. As shown in Figure 1A, after visualization by in-gel protein staining, there was a single band in each lane near 63-66 kD which was close to the molecular weight of M13KE (about 63 kD) according to the protein ladder.

### Detection of B and T cell epitopes

B cell epitope was confirmed by Western blot assay as described in the methods. As shown in Figure 1B and 1C, all recombinant phages containing epitopes of OmpL1 or LipL41 reacted with the serum against leptospire (*L. interrogans *strain 56601), rOmpL1 and rLipL41. Through quantitative analysis using quantity one 4.6.3 software (Bio-Rad), we found that there were differences in the reactivity among the anti-sera of recombinant proteins and leptospire. The band representing OmpL1 residues 173-191 (OmpL1_173-191_) showed most significant reactivity with anti-rOmpL1 serum, and OmpL1_297-320 _was more reactive than the rest two epitopes. All the four recombinant phages reacted with the anti-leptospire serum. Phages containing OmpL1_87-98 _reacted most significantly. The reactivity of phages containing OmpL1_59-78 _and phages containing OmpL1_297-320 _was close. When the phage particles were incubated with anti-rLipL41 serum, the reactivity of phages containing epitope LipL41_181-195 _or LipL41_263-282 _was more remarkable than phages containing the other two epitopes. When incubating with anti-leptospire serum, the reactivity of phages containing LipL41_233-256 _was the lowest comparing to the other three epitopes. Five anti-leptospire sera from leptospire-infected humans were pooled together to test the reactivity against each B cell epitope. The result showed that epitope OmpL1_87-98 _reacted the strongest among the four OmpL1 epitopes, and LipL41_233-256 _was the lowest among the four LipL41 epitopes (Figure 1D).

**Figure 2:**
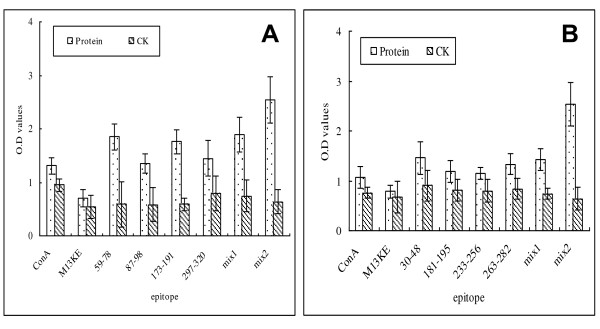
**Proliferation rate of epitopes stimulated splenocytes**. 5 × 10^4 ^splenocytes and 10^5 ^mitomycin-treated cells were mixed and stimulated with phage particles containing epitopes of OmpL1 (A) or LipL41 (B) to test the proliferation of the cells. Response to each antigen was presented as the mean value of three independent experiments. Splenocytes were isolated from PBS control mice to determine if the responses were OmpL1- or LipL41-specific. The cells stimulated with ConA and wild-type phages were used as controls. The data were representative of three independent experiments. Mix1 stands for the data from the epitope mixture of OmpL1 or LipL41 stimulating splenocytes from OmpL1- or LipL41-immunized mice. Mix2 stand for the data from the epitope mixture of both OmpL1 and LipL41 stimulating the splenocytes from OmpL1- or LipL41- immunized mice.

T cell epitope was examined using proliferation assay of CD4^+ ^T cells. As shown in Figure 2, in comparison with that from PBS control mice, splenocytes harvested from rOmpL1- or rLipL41-immunized mice proliferated vigorously upon stimulation with phages expressing epitope peptides of OmpL1 or LipL41.

Haake and his coworkers [16] previously reported that OmpL1 and LipL41 exhibited synergistic immunoprotection in Golden Syrian hamster model. To determine if there are also synergistic interactions among the epitope peptides, phages expressing each peptide of OmpL1 and LipL41 were pooled together and were used to stimulate the splenocytes. As shown in Figure 2, proliferation of splenocytes stimulated with the 8-epitope mixture (mix2) was more significant comparing to single-epitope phages or 4-epitope mixture of OmpL1 or LipL41 alone (mix1).

### Evaluation of cytokine secretion in splenocytes induced by OmpL1- or LipL41-derived epitopes

**Figure 3:**
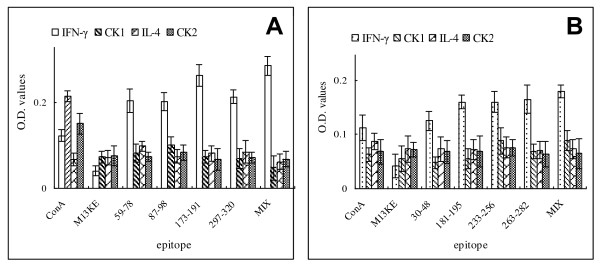
**Cytokine profiles of T cells from mice spleen**. Splenocytes from recombinant OmpL1 (A) or LipL41 (B) immunized mice were isolated 10 days after the last immunization and were stimulated with epitopes from corresponding proteins in vitro for 72 hours. Mix stand for the data from the epitope mixture of OmpL1 or LipL41 stimulating the splenocytes from OmpL1- or LipL41- immunized mice. Each value is representative of 3 mice in triplicates.

ELISA assay was employed to determine the *in vitro *polarization of T helper cells. Cells from both OmpL1- and LipL41-immunized mice released large amount of IFN-γ but not IL-4 comparing to cells from PBS control mice (Figure 3). OmpL1_173-191 _epitope showed the strongest activity of stimulation, and other three OmpL1 epitopes showed similar abilities in the stimulation of IFN-γ secretion. Among the LipL41 epitopes, the secretion of IFN-γ in the cell cultures was induced by LipL41_181-195_, LipL41_233-256 _and LipL41_263-282 _to the similar level; all of them were stronger than LipL41_30-48_. When the 4 epitopes of OmpL1 were pooled together to stimulate the splenocytes, the secretion of IFN-γ cytokine in the splenocyte supernatants was mildly increased. Phages expressing each epitope of LipL41 failed to stimulate the secretion of IFN-γ or IL-4 (Figure 3B).

## Discussion

*Leptospira interrogans *causes disease in both animals and humans throughout the world. Leptospirosis in humans may be fatal due to the involvement of severe damage to multiple organs such as liver, lung, kidney and brain and is an increasing concern to the public health [24].

*L. interrogans *can rapidly disseminate to multiple organs to induce programmed cell death [25,26]. The essential properties of a vaccine are safe, immunogenic, and effective in the prevention of leptospiral infection at both acute and carrier state. It has been a challenge to develop an effective and safe *L. interrogans *vaccine [27]. The currently available vaccines include multiple-valence inactivated leptospiral vaccine and subunit leptospiral vaccines [28]. However, these vaccines often have serious adverse effects [29]. And more importantly, most recombinant protein vaccines used against *Leptospira *in animals are serovar-specific and therefore their efficacy is limited when *Leptospira *of a different serovar is circulating [30]. The current emphasis in research laboratories is to discover conserved antigens that may induce long term protection across the species or serovars of *Leptospira*. A better understanding of the mechanisms of the humoral as well as cell-mediated immune responses that are protective during leptospirosis of animals or human is critical to the development of a good leptospiral vaccine.

Epitopes are small particular segments in antigen molecules which usually affect the antigenic specificity of the cellular and humoral immune responses. Epitopes are attracting in the development of leptospiral vaccines, because it is convenient to make a seasonal vaccine by simply changing the formulations of the epitopes of relevant species. There are two types of epitopes of antigens, linear and conformational. A linear or a sequential epitope is an epitope that is recognized by the antibodies by its linear sequence of amino acids, and a conformational epitope is the sequences of subunits composing an antigen (usually amino acids of a protein antigen) that directly binds to a receptor of the immune system [31,32]. Commonly, T cell epitopes are regulated by antigen presenting cells (APC) to induce cellular immune response [20]. B cell epitopes including linear and conformational structure mostly induce humoral immune response [33,34]. Multi-epitope peptide vaccine has become an attractive strategy in the development of vaccines against pathogens. Usually, a good epitope vaccine contains both B cell and T cell epitopes.

*in silico *epitope prediction is a useful tool in the development of new vaccine formulations [35-37]. We set out to identify combined T and B cell epitopes of the leptospiral outer membrane proteins which are closely associated with leptospirosis. *In silico *epitope prediction led to the identification of four combined T and B cell epitopes of OmpL1 and four combined T and B cell epitopes of LipL41, respectively. The predicted epitopes were distributed along the entire protein sequence of each outer membrane protein. We compared the sequences of these epitopes to the sequences of 15 official Chinese standard strains, and found that all the epitopes were identical to the corresponding regions of OmpL1 and LipL41 of these different *Leptospira *strains. To confirm B cell epitopes, we used phage display system and Western blot analysis which were efficient methods in the study of B cell epitopes [38]. Our result showed that these selected epitopes were specifically recognized by the antibodies in the rabbit sera against *Leptospira interrogans*, rOmpL1 or LipL41 but the reactivity of each epitope to the antibodies was different. We speculate that this might be due to the interference of the recognition by the recombinant proteins still present in the sera. Pathogenic microorganisms induce humoral immune responses during infection, which specifically responses to the antigens through specific interactions between the antibodies and the epitopes of the antigens [39]. Recognition of the epitopes by antisera from immunized BALB/c mice was confirmed, suggesting that these epitopes displayed by phages resemble the ones in the native antigen protein. In this study, we have shown that T and B cell combined epitopes of leptospiral outer membrane proteins OmpL1 and LipL41 induced leptospire-specific immune responses, which suggests that these epitopes instead of entire proteins may be used to develop leptospiral vaccines.

CD4^+ ^T lymphocytes play a critical role in the host immune responses during bacterial infection [40,41]. CD4^+ ^T cells have been shown to differentiate into Th1, Th2 and lately Th17 (important to intracellular bacteria) cells. Th1 cells are characterized by their production of IFN-γ and are involved in cellular immunity [42,43], and Th2 cells produce IL-4 and are required for humoral immunity [44]. In this experiment, the secretion of IFN-γ was more distinct than that of IL-4 when the splenocytes were stimulated with the epitopes. We did not detect any significant secretion of IL-4 in epitope-stimulated splenocyte cultures. It is possible that the levels of IL-4 were below detection limit. The results implied that the selected epitopes were BALB/c-specific Th1-type epitope. Immune protection against leptospires in mice is primarily correlated with Th1-mediated immunity and IFN-γ secretion [45]. This result is consistent with our previous findings on *Leptospira *antigens LipL32 and LipL21 proteins[22], suggesting that epitopes of outer membrane proteins (eg, OmpL1, LipL21, LipL32 and LipL41) can induce strong cell-mediated immune response as well humoral immune responses. These epitopes may help us to investigate the role of Th1 or Th2 responses in the pathogenesis and immunity during *Leptospira interrogans *infection.

## Conclusions

The Western blot data present here indicated that the combined T and B cells epitopes in outer membrane proteins of *L. interrogans *can be recognized by antibodies in the sera from leptospire-infected patients or rabbits immunized with recombinant proteins of outer membrane proteins. The data from T cell proliferation assay and cytokines secretion analysis showed that the selected epitopes can induce a Th1- orientated response. The present study revealed that peptides OmpL1_173-191 _of OmpL1 and LipL41_233-256 _of LipL41 are both T cell and B cell epitopes which collaborate in the production of antibodies against leptospire and induction of lymphocyte differentiation. The identification of these immune dominant epitopes may greatly facilitate the development of novel leptospiral vaccines which may provide protections across different serogroups or serovars.

## Authors' contributions

LXA designed the work, performed the research study, and prepared the manuscript. SAH and RP participated in all experimental work. ZZ was involved in the revision of the manuscript. YJ designed and supervised the research study. All authors read and approved the final version of the manuscript.

## Supplementary Material

Additional file 1 **PCR amplification of epitopes**. Predicted epitope fragments of OmpL1 and LipL41 were amplified from genomic DNA of Lai strain. M is the DNA ladder. 1-4 are the epitope fragments 59-78, 87-98, 173-191 and 297-320 of OmpL1. 5-8 are the epitope fragments 30-48, 181-195, 233-256 and 263-282 of LipL41. Click here for file

Additional file 2 **PCR confirmation of epitope insertion in the recombinant phage**. The inserted epitope fragment in recombinant M13KE was confirmed by colony PCR. M is the DNA ladder. 1 is the fragment amplified from wild type phage M13KE, 2-5 are the epitope fragments 59-78, 87-98, 173-191 and 297-320 of OmpL1. 6-9 are the epitope fragments 30-48, 181-195, 233-256 and 263-282 of LipL41. Click here for file

## References

[B1] McBride AJ Athanazio DA Reis MG Ko AI Leptospirosis Curr Opin Infect Dis 2005 18 5 376 386 10.1097/01.qco.0000178824.05715.2c 16148523

[B2] Palaniappan RU Ramanujam S Chang YF Leptospirosis: pathogenesis, immunity, and diagnosis Curr Opin Infect Dis 2007 20 3 284 292 10.1097/QCO.0b013e32814a5729 17471039

[B3] Lindenstrøm T Agger EM Korsholm KS Darrah PA Aagaard C Seder RA Rosenkrands I Andersen P Tuberculosis subunit vaccination provides long-term protective immunity characterized by multifunctional CD4 memory T cells J Immunol 2009 182 12 8047 8055 10.4049/jimmunol.0801592 19494330

[B4] Naiman BM Alt D Bolin CA Zuerner R Baldwin C Protective killed Leptospira borgpetersenii vaccine induces potent Th1 immunity comprising responses by CD4 and gammadelta T lymphocytes Infect Immun 2001 69 7550 7558 10.1128/IAI.69.12.7550-7558.2001 11705932 PMC98846

[B5] Srinivasan A Nanton M Griffin A McSorley SJ Culling of activated CD4 T cells during typhoid is driven by *Salmonella *virulence genes J Immunol 2009 182 12 7838 7845 10.4049/jimmunol.0900382 19494308 PMC2731968

[B6] Faine S Adler B Bolin C Perolat P Pathogenesis, virulence, immunity Leptospira and Leptospirosis 1999 2 MediSci, Melbourne, Vic. Australia 73 91

[B7] Nascimento AL Ko AI Martins EA Monteiro-Vitorello CB Ho PL Haake DA Verjovski-Almeida S Hartskeerl RA Marques MV Oliveira MC Menck CF Leite LC Carrer H Coutinho LL Degrave WM Dellagostin OA El-Dorry H Ferro ES Ferro MI Furlan LR Gamberini M Giglioti EA Góes-Neto A Goldman GH Goldman MH Harakava R Jerônimo SM Junqueira-de-Azevedo IL Kimura ET Kuramae EE Lemos EG Lemos MV Marino CL Nunes LR de Oliveira RC Pereira GG Reis MS Schriefer A Siqueira WJ Sommer P Tsai SM Simpson AJ Ferro JA Camargo LE Kitajima JP Setubal JC Van Sluys MA Comparative genomics of two *Leptospira interrogans *serovars reveals novel insights into physiology and pathogenesis J Bacteriol 2004 186 7 2164 2172 10.1128/JB.186.7.2164-2172.2004 15028702 PMC374407

[B8] Ren SX Fu G Jiang XG Zeng R Miao YG Xu H Zhang YX Xiong H Lu G Lu LF Jiang HQ Jia J Tu YF Jiang JX Gu WY Zhang YQ Cai Z Sheng HH Yin HF Zhang Y Zhu GF Wan M Huang HL Qian Z Wang SY Ma W Yao ZJ Shen Y Qiang BQ Xia QC Guo XK Danchin A Saint Girons I Somerville RL Wen YM Shi MH Chen Z Xu JG Zhao GP Unique physiological and pathogenic features of *Leptospira interrogans *revealed by whole-genome sequencing Nature 2003 422 6934 888 893 10.1038/nature01597 12712204

[B9] Bulach DM Zuerner RL Wilson P Seemann T McGrath A Cullen PA Davis J Johnson M Kuczek E Alt DP Peterson-Burch B Coppel RL Rood JI Davies JK Adler B Genome reduction in *Leptospira borgpetersenii *reflects limited transmission potential Proc Natl Acad Sci USA 2006 103 39 14560 14565 10.1073/pnas.0603979103 16973745 PMC1599999

[B10] Picardeau M Bulach DM Bouchier C Zuerner RL Zidane N Wilson PJ Creno S Kuczek ES Bommezzadri S Davis JC McGrath A Johnson MJ Boursaux-Eude C Seemann T Rouy Z Coppel RL Rood JI Lajus A Davies JK Médigue C Adler B Genome sequence of the saprophyte *Leptospira biflexa *provides insights into the evolution of *Leptospira *and the pathogenesis of leptospirosis PLoS One 2008 3 2 e1607 10.1371/journal.pone.0001607 18270594 PMC2229662

[B11] Cullen PA Haake DA Adler B Outer membrane proteins of pathogenic spirochetes FEMS Microbiol Rev 2004 28 3 291 318 10.1016/j.femsre.2003.10.004 15449605 PMC2666356

[B12] Haake DA Champion CI Martinich C Shang ES Blanco DR Miller JN Lovett MA Molecular cloning and sequence analysis of the gene encoding OmpL1, a transmembrane outer membrane protein of pathogenic *Leptospira spp* J Bacteriol 1993 175 13 4225 4234 8320237 10.1128/jb.175.13.4225-4234.1993 PMC204853

[B13] Shang ES Summers TA Haake DA Molecular cloning and sequence analysis of the gene encoding LipL41, a surface-exposed lipoprotein of pathogenic *Leptospira *species Infect Immun 1996 64 6 2322 2330 8675344 10.1128/iai.64.6.2322-2330.1996 PMC174073

[B14] Dong H Hu Y Xue F Sun D Ojcius DM Mao Y Yan J Characterization of the ompL1 gene of pathogenic *Leptospira *species in China and cross-immunogenicity of the OmpL1 protein BMC Microbiol 2008 8 223 10.1186/1471-2180-8-223 19087358 PMC2632671

[B15] Guerreiro H Croda J Flannery B Mazel M Matsunaga J Galvão Reis M Levett PN Ko AI Haake DA Leptospiral proteins recognized during the humoral immune response to leptospirosis in humans Infect Immun 2001 69 8 4958 4968 10.1128/IAI.69.8.4958-4968.2001 11447174 PMC98588

[B16] Haake DA Mazel MK McCoy AM Milward F Chao G Matsunaga J Wagar EA Leptospiral outer membrane proteins OmpL1 and LipL41 exhibit synergistic immunoprotection Infect Immun 1999 67 12 6572 6582 10569777 10.1128/iai.67.12.6572-6582.1999 PMC97069

[B17] Ding W Yan J Mao YF Genotyping of LipL41 genes from *Leptospira interrogans *serogroups and immunological identification of the expression products Chin J Microbiol Immunol 2004 24 11 859 865

[B18] Xu Y Yan J Mao YF Li LW Li SP Genotypes of the OmpL1 gene from the dominant serogroups of *Leptospira interrogans *in China and construction of prokaryotic expression system of the gene and immunological identification of the recombinant protein Chin J Microbiol Immunol 2004 24 6 439 444

[B19] Lin X Chen Y Yan J Recombinant multiepitope protein for diagnosis of leptospirosis Clin Vaccine Immunol 2008 15 11 1711 1714 10.1128/CVI.00189-08 18827193 PMC2583528

[B20] Singh H Raghava GPS ProPred: Prediction of HLA-DR binding sites Bioinformatics 2001 17 12 1236 1237 10.1093/bioinformatics/17.12.1236 11751237

[B21] Lin X Chen Y Lu Y Yan J Yan J Application of a loop-mediated isothermal amplification method for the detection of pathogenic *Leptospira* Diagn Microbiol Infect Dis 2009 63 3 237 242 10.1016/j.diagmicrobio.2008.10.012 19070450

[B22] Lin X Zhao J Qian J Mao Y Pan J Li L Peng H Luo Y Yan J Identification of immunodominant B- and T-cell combined epitopes in outer membrane lipoproteins LipL32 and LipL21 of *Leptospira interrogans* Clin Vaccine Immunol 2010 17 5 778 783 10.1128/CVI.00405-09 20237196 PMC2863375

[B23] Day LA Conformations of single-stranded DNA and coat protein in fd bacteriophage as revealed by ultraviolet absorption spectroscopy J Mol Biol 1969 39 2 265 277 10.1016/0022-2836(69)90316-7 5362671

[B24] Dutta TK Christopher M Leptospirosis-an overview J Assoc Physicians India 2005 53 545 551 16121811

[B25] Barocchi MA Ko AI Reis MG McDonald KL Riley LW Rapid translocation of polarized MDCK cell monolayers by *leptospira *ubterrigabs, an invasive but nonintracellular pathogen Infect Immun 2002 70 12 6926 6932 10.1128/IAI.70.12.6926-6932.2002 12438371 PMC132952

[B26] Jin D Ojcius DM Sun D Dong H Luo Y Mao Y Yan J *Leptospira interrogans *induces apoptosis in macrophages via caspase-8- and caspase-3 dependent pathways Infect Immun 2009 77 2 799 809 10.1128/IAI.00914-08 19029301 PMC2632035

[B27] Gordon PJ Control of leptospirosis by vaccination Vet Rec 2002 150 13 420 11999287

[B28] Wang Z Jin L Wegrzyn A Leptospirosis vaccines Microb Cell Fact 2007 6 1 39 10.1186/1475-2859-6-39 18072968 PMC2231387

[B29] Thongboonkerd V Proteomics in leptospirosis research: towards molecular diagnostics and vaccine development Expert Rev Mol Diagn 2008 8 1 53 61 10.1586/14737159.8.1.53 18088230

[B30] Sonrier C Branger C Michel V Ruvoen-Clouet N Ganiere JP Andre-Fontaine G Evidence of crossprotection within *Leptospira interrogans *in an experimental model Vaccine 2000 19 1 86 94 10.1016/S0264-410X(00)00129-8 10924790

[B31] Goldsby R Kindt TJ Osborne BA Janis K Antigens Immunology 2003 5 New York: W. H. Freeman and Company 57 75

[B32] Wang LF Yu M Epitope identification and discovery using phage display libraries: applications in vaccine development and diagnostics Curr Drug Targets 2004 5 1 1 15 10.2174/1389450043490668 14738215

[B33] Conway JF Watts NR Belnap DM Cheng N Stahl SJ Wingfield PT Steven AC Characterization of a conformational epitope on hepatitis B virus core antigen and quasiequivalent variations in antibody binding J Virol 2003 77 11 6466 6473 10.1128/JVI.77.11.6466-6473.2003 12743303 PMC155010

[B34] Malm M Rollman E Ustav M Hinkula J Krohn K Wahren B Blazevic V Cross-clade protection induced by human immunodeficiency virus-1 DNA immunogens expressing consensus sequences of multiple genes and epitopes from subtypes A, B, C, and FGH Viral Immunol 2005 18 4 678 688 10.1089/vim.2005.18.678 16359234

[B35] Fonseca CT Cunha-Neto E Kalil J Jesus AR Correa-Oliveira R Carvalho EM Oliveira SC Identification of immunodominant epitopes of *Schistosoma mansoni *vaccine candidate antigens using human T cells Mem Inst Oswaldo Cruz 2004 99 5 Suppl 1 63 66 15486637 10.1590/s0074-02762004000900011

[B36] Iwai LK Yoshida M Sidney J Shikanai-Yasuda MA Goldberg AC Juliano MA Hammer J Juliano L Sette A Kalil J Travassos LR Cunha-Neto E In silico prediction of peptides binding to multiple HLA-DR molecules accurately identifies immunodominant epitopes from gp43 of *Paraccocidioides brazilienses *frequently recognized in primary peripheral blood mononuclear cell responses from sensitized individuals Mol Med 2003 9 9-12 209 219 15208742 PMC1430984

[B37] Panigada M Sturniolo T Besozzi G Boccieri MG Sinigaglia F Grassi GG Grassi F Identification of a promiscuous T cell epitope in *Mycobacterium tuberculosis *Mce proteins Infect Immun 2002 70 1 79 85 10.1128/IAI.70.1.79-85.2002 11748166 PMC127636

[B38] Rowley MJ O'Connor K Wijeyewickrema L Phage display for epitope determination: a paradigm for identifying receptor-ligand interactions Biotechnol Annu Rev 2004 10 151 188 full_text 15504706 10.1016/S1387-2656(04)10006-9

[B39] Gershoni JM Roitburd-Berman A Siman-Tov DD Tarnovitski Freund N Weiss Y Epitope mapping: the first step in developing epitope-based vaccines BioDrugs 2007 21 3 145 156 10.2165/00063030-200721030-00002 17516710 PMC7100438

[B40] Chinen J Shearer WT Basic and clinical immunology J Allergy Clin Immunol 2005 116 2 411 418 10.1016/j.jaci.2005.05.010 16083798

[B41] Haque A Blum JS New insights in antigen processing and epitope selection: development of novel immunotherapeutic strategies for cancer, autoimmunity and infectious diseases J Biol Regul Homeost Agents 2005 19 3-4 93 104 16602623

[B42] Schroder K Hertzog PJ Ravasi T Hume DA Interferon-gamma: an overview of signals, mechanisms and functions J Leukoc Biol 2004 75 2 163 189 10.1189/jlb.0603252 14525967

[B43] Kita M Role of IFN-gamma in nonviral infection Nippon Rinsho 2006 64 7 1269 1274 16838643

[B44] Zhou L Chong MM Littman DR Plasticity of CD4+ T cell lineage differentiation Immunity 2009 30 5 646 655 10.1016/j.immuni.2009.05.001 19464987

[B45] Vernel-Pauillac F Merien F Proinflammatory and immunomodulatory cytokine mRNA time course profiles in hamsters infected with a virulent variant of *Leptospira interrogans* Infect Immun 2006 74 7 4172 4179 10.1128/IAI.00447-06 16790792 PMC1489750

