# Supplementary figures and images for: Characterization of Conserved Combined T and B Cell Epitopes in Leptospira interrogans Major Outer Membrane Proteins OmpL1 and LipL41
Source: BMC Microbiol. 2011 Jan 26;11:21. doi: 10.1186/1471-2180-11-21 (PMC3038132; doi:10.1186/1471-2180-11-21)

## Slide 1
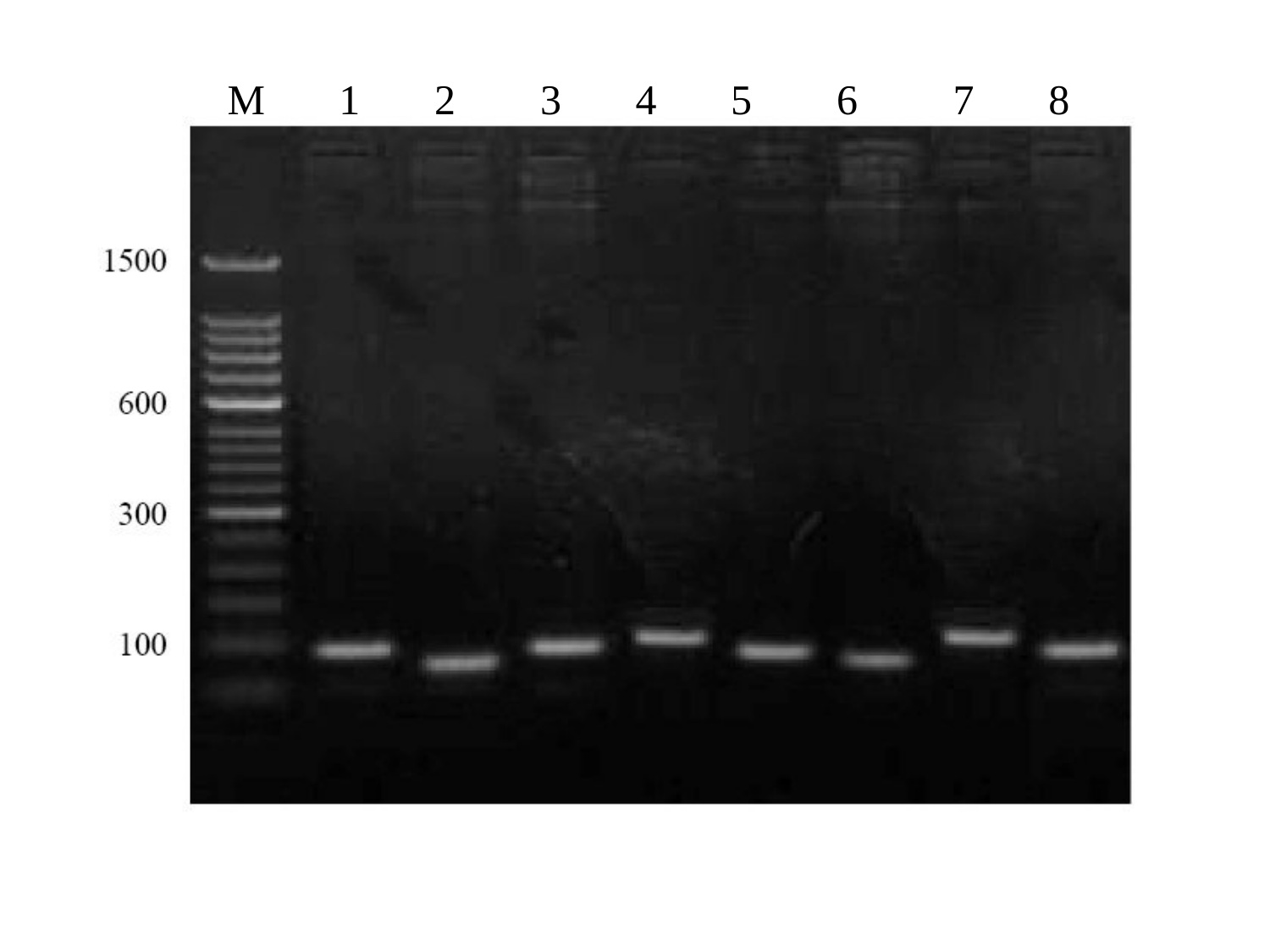

M 1 2 3 4 5 6 7 8

Supplement: Additional file 1 — PCR amplification of epitopes. Predicted epitope fragments of OmpL1 and LipL41 were amplified from genomic DNA of Lai strain. M is the DNA ladder. 1-4 are the epitope fragments 59-78, 87-98, 173-191 and 297-320 of OmpL1. 5-8 are the epitope fragments 30-48, 181-195, 233-256 and 263-282 of LipL41. [file 1471-2180-11-21-S1.PPT]

## Slide 1
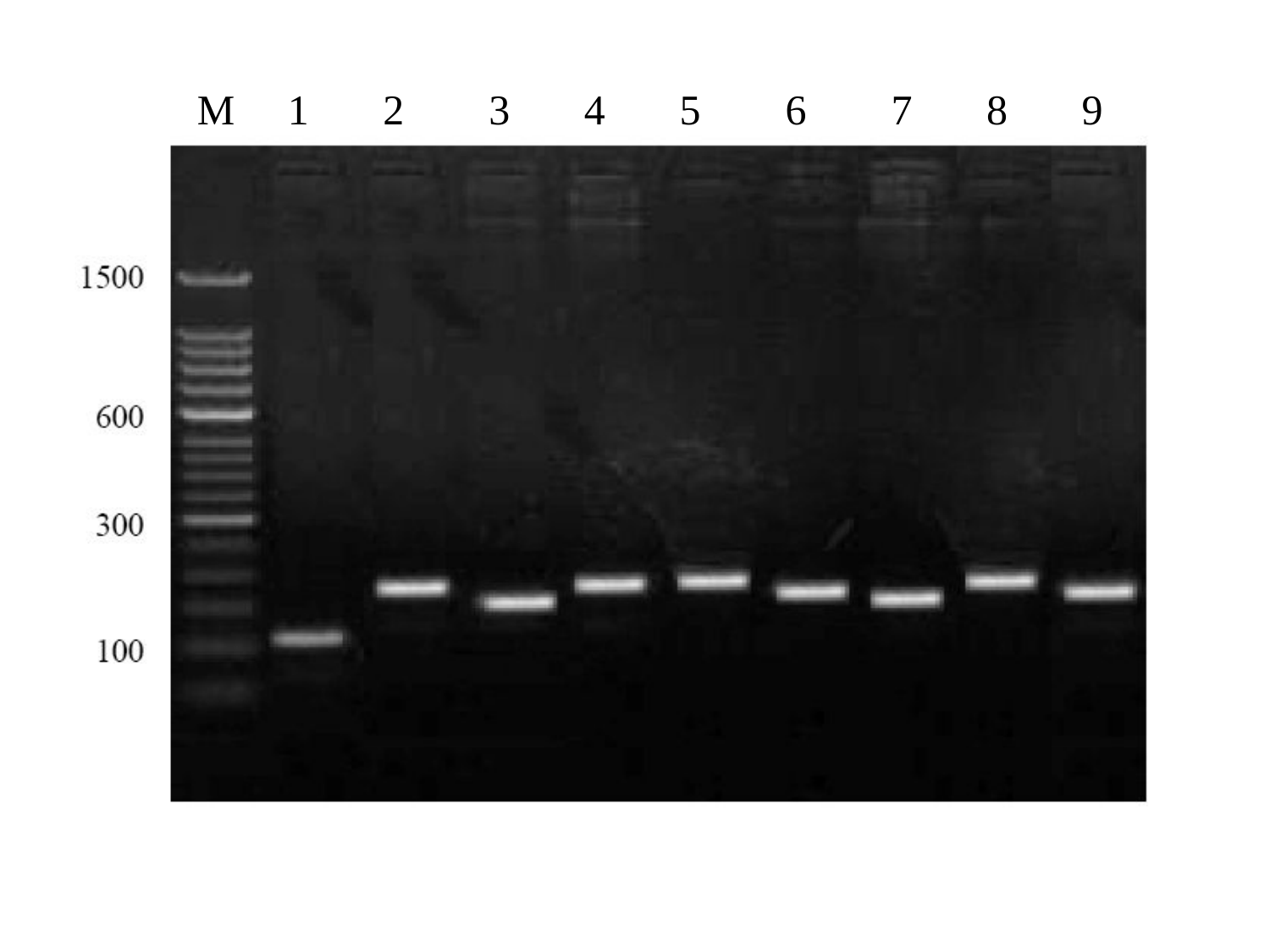

M 1 2 3 4 5 6 7 8 9

Supplement: Additional file 2 — PCR confirmation of epitope insertion in the recombinant phage. The inserted epitope fragment in recombinant M13KE was confirmed by colony PCR. M is the DNA ladder. 1 is the fragment amplified from wild type phage M13KE, 2-5 are the epitope fragments 59-78, 87-98, 173-191 and 297-320 of OmpL1. 6-9 are the epitope fragments 30-48, 181-195, 233-256 and 263-282 of LipL41. [file 1471-2180-11-21-S2.PPT]
